# Small-Molecule Compounds Boost CAR-T Cell Therapy in Hematological Malignancies

**DOI:** 10.1007/s11864-023-01049-4

**Published:** 2023-01-26

**Authors:** Xinping Cao, Xin Jin, Xiaomei Zhang, Paudel Utsav, Yi Zhang, Ruiting Guo, Wenyi Lu, Mingfeng Zhao

**Affiliations:** 1 First Center Clinic College of Tianjin Medical University, Tianjin, 300192 China; 2 Department of Hematology, Tianjin First Central Hospital, Tianjin, 300192 China; 3 School of Medicine, Nankai University, Tianjin, 300071 China

**Keywords:** Chimeric antigen receptor T cells, Hematological malignancies, Small-molecule compounds

## Abstract

Although chimeric antigen receptor T cell immunotherapy has been successfully applied in patients with hematological malignancies, several obstacles still need to be overcome, such as high relapse rates and side effects. Overcoming the limitations of CAR-T cell therapy and boosting the efficacy of CAR-T cell therapy are urgent issues that must be addressed. The exploration of small-molecule compounds in combination with CAR-T cell therapies has achieved promising success in pre-clinical and clinical studies in recent years. Protein kinase inhibitors, demethylating drugs, HDAC inhibitors, PI3K inhibitors, immunomodulatory drugs, Akt inhibitors, mTOR inhibitors, and Bcl-2 inhibitors exhibited potential synergy in combination with CAR-T cell therapy. In this review, we will discuss the recent application of these combination therapies for improved outcomes of CAR-T cell therapy.

**Supplementary Information:**

The online version contains supplementary material available at 10.1007/s11864-023-01049-4.

## Introduction

**Fig. 1:**
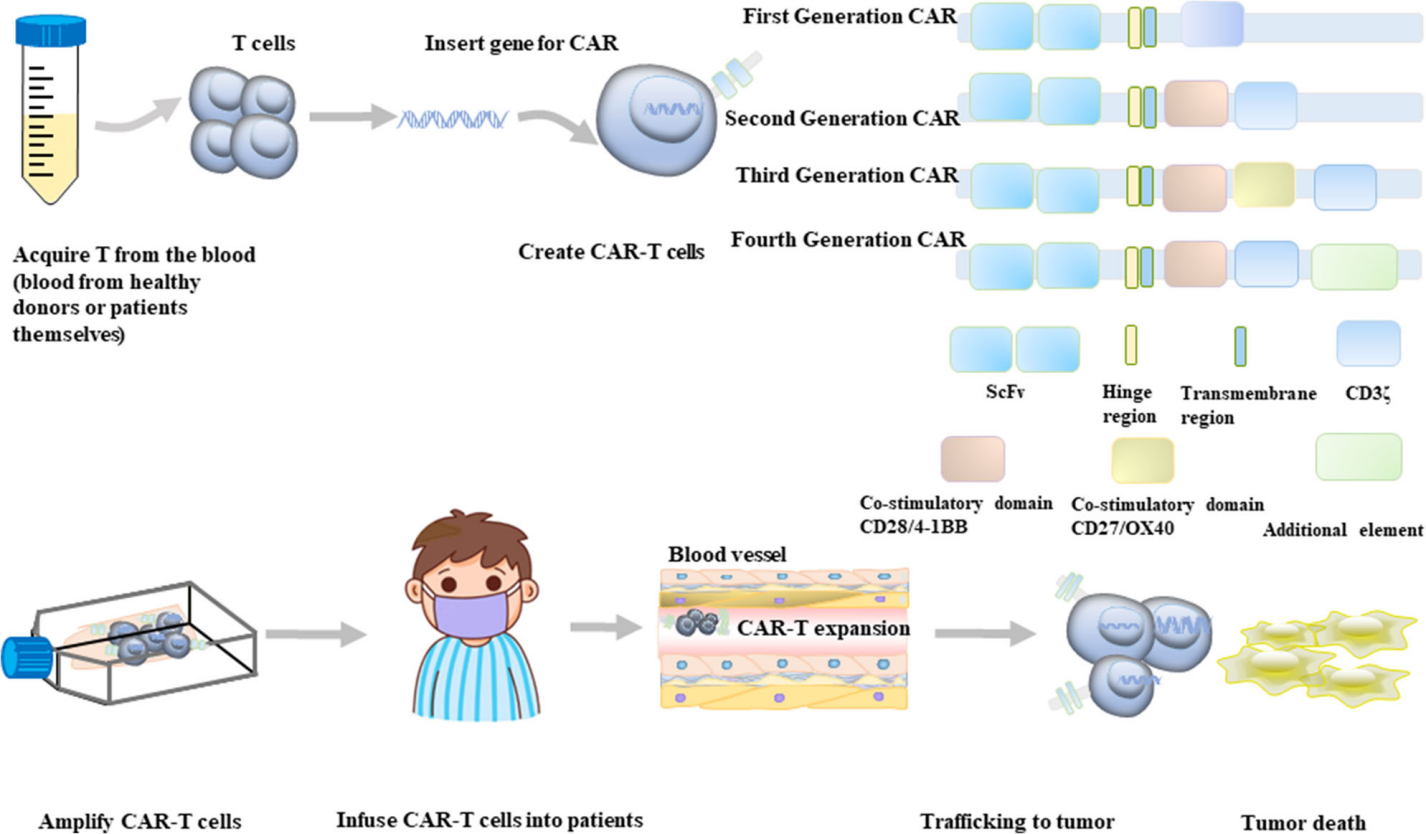
The process of CAR-T cell therapy. (1) Acquiring T cells from patients’ or healthy donors’ blood; (2) Create CAR-T cells: T cells are genetically engineered into CAR-T cells. CARs are artificial proteins, consisting of an extracellular antigen recognition region (single-chain variable fragment (ScFv)), a hinge domain, a transmembrane region, and an intracellular signal transduction region (zero to three costimulatory domains, and an intracellular CD3ζ activation region). According to the different structures of the intracellular signal transduction region, it can be categorized into four generations, and the basic structure includes ScFv, a hinge domain, a transmembrane region, and CD3ζ. The first generation only has the basic structure. The second generation adds a costimulatory part (CD28 or 4-1BB). The third generation obtains two or more costimulatory parts (CD28, 4-1BB, ICOS, OX-40, etc.). The fourth generation contains additional protein molecules or gets extra receptors. (3) Amplify CAR-T cells; (4) Infusion CAR-T cells into patients: CAR-T cells are engrafted in patients and increase widely. Meanwhile, many tumor cells can be killed by one CAR-T cell. CAR-T cells can facilitate immune surveillance, prevent tumor recurrence, and assist tumor-infiltrating lymphocytes in attacking tumors through antigen release or the CAR-T cells’ persistence.

In such an era where immunotherapy develops rapidly, CAR-T cell therapy has remarkably succeeded in treating patients with hematologic malignancies, especially suffering from relapsed/refractory B cell acute lymphoblastic leukemia (r/r B-ALL) [1]. T cells are usually isolated from patients or suitable donors, and then these cells are genetically engineered into CAR-T cells, which can especially recognize and kill tumor cells in a non-MHC-restricted manner [2]. CARs are artificial proteins including four sections: an extracellular antigen recognition domain, a hinge region, a transmembrane domain, and an intracellular signal transduction domain. Conventionally, according to the composition of the intracellular signal transduction region, CAR-T cells are categorized into four generations [3] (Fig. 1). The first generation has only one intracellular activation domain named CD3ζ, an immunoreceptor tyrosine-activated motif (ITAM), triggering the downstream sequences after the activation. However, due to the lack of the costimulatory signal domain, the levels of cytokine secretion are low. Therefore, the anti-tumor effects are equally inferior. Owing to the poor persistence, the first generation of CAR-T has little application in clinical practices [4]. The second generation has one costimulatory signaling domain (CD28 or 4-1BB). A large number of studies have shown that this design increases memory effects of tumor cell lysis and the lethal effects mediated by CAR-T cells. In addition, the persistence of its impacts is also improved compared to its initial generation [5]. The third generation possesses two or more costimulatory signaling regions (CD28, 4-1BB, ICOS, OX-40, etc.), so T cells can produce more cytokines and exert more robust and durable anti-tumor effects after activation. The fourth generation of CAR-T cells is sometimes also called TRUCK T cells (T cell redirected for universal cytokine killing). It contains additional protein molecules such as interleukin-12 or extra receptors such as costimulatory ligands to regulate the tumor microenvironment (TME) and recruit and activate other immune cells for tumor killing [6]. At present, six CAR-T cell products targeting CD19 or BCMA have been launched worldwide by the FDA. These products all pertain to the second generation. Existing studies have demonstrated that for patients with r/r B-ALL, the complete remission (CR) rate of CAR-T cell therapy already reaches 70 to 90% [2, 7]. The CR rate of r/r lymphoma patients is 50 to 67% [8–10], and the remission rate in multiple myeloma (MM) can reach more than 80% [11, 12].

**Fig. 2:**
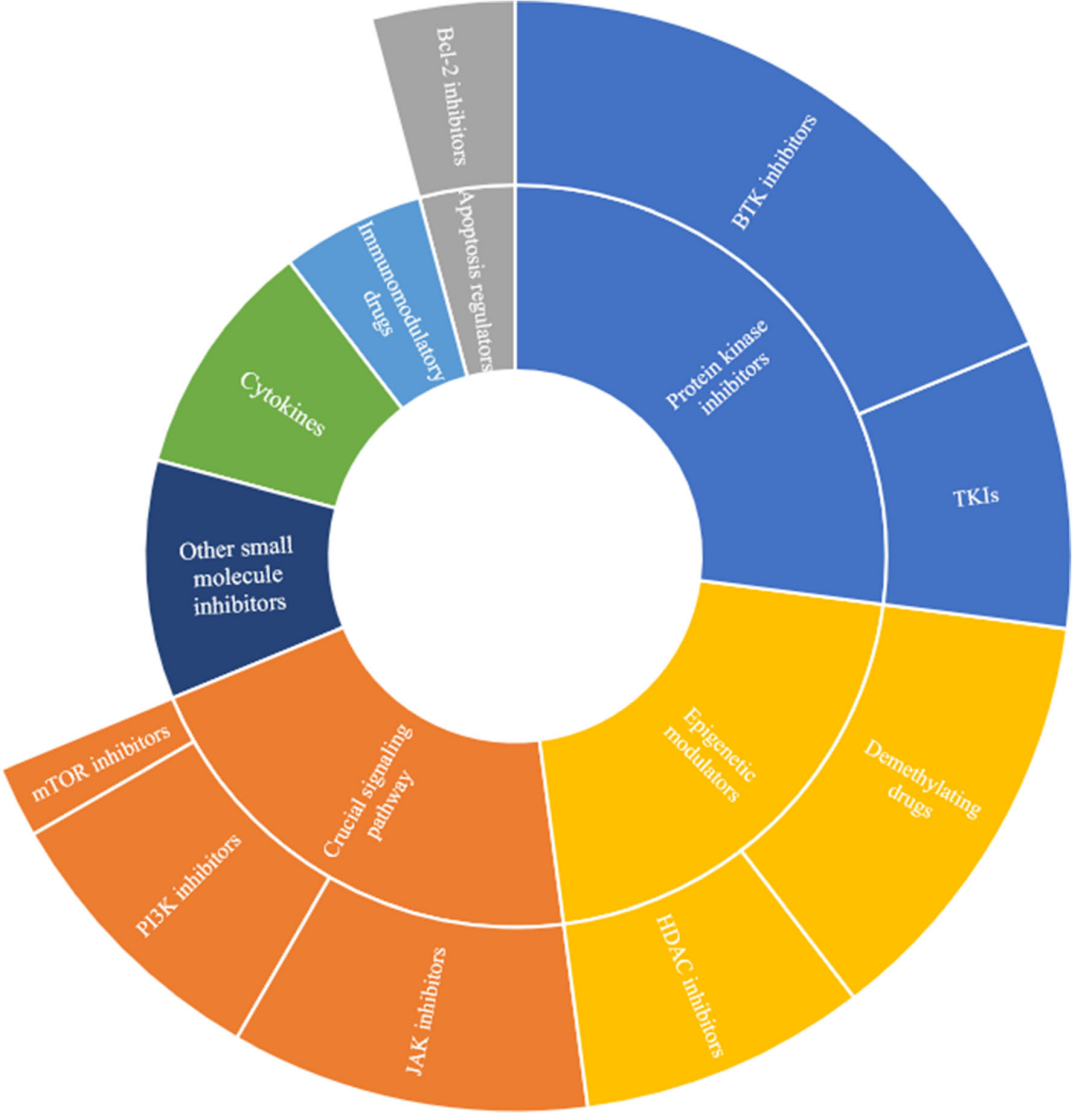
CAR-T cell therapy in combination with different kinds of small-molecule compounds. TKIs, tyrosine kinase inhibitors; BTK, Bruton tyrosine kinase; JAK, Janus kinase; PI3K, phosphoInositide-3 kinase; Bcl–2, B cell lymphoma-2; HDAC, histone deacetylase.

However, with the further application and the deepening research of CAR-T cell therapy, problems such as resistance, recurrences, and toxicity have gradually emerged [13, 14]. In combination with the existing drugs that are applied to hematological malignancies, patients with a poor initial response to CAR-T cell therapy or patients with disease recurrence after CAR-T cell therapy have found new hope. At present, small-molecule compounds can mitigate the limitations of CAR-T cell therapy, including the expression of inhibitory receptors, poor amplification, inferior persistence, loss of target antigen, severe cytokine release syndrome (CRS), or neurotoxicity, etc. [15••]. These compounds include small-molecule inhibitors, such as tyrosine kinase inhibitors (TKIs), Bruton tyrosine kinase (BTK) inhibitors, Janus kinase (JAK) inhibitors, phosphoInositide-3 kinase (PI3K) inhibitors, mammalian target of rapamycin (mTOR) inhibitors, B cell lymphoma-2 (Bcl-2) inhibitors, histone deacetylases (HDAC) inhibitors, cox-2 inhibitors, nuclear output selective inhibitors, gamma-secretase inhibitors, etc., small molecules including demethylation drugs, immunomodulators, and cytokines, and some other cytokine-blocking antibodies, immune checkpoint inhibitors, monoclonal antibodies, and chemotherapy drugs, etc. The purpose of this article is to summarize and briefly analyze the research progress of CAR-T cell immunotherapy combined with small-molecule compounds to treat hematological malignancies (Fig. 2).

## Protein kinase inhibitors

Protein phosphorylation is a critical step in cell growth and is essential for maintaining cellular homeostasis. Meanwhile, protein kinases are the key enzymes that promote protein phosphorylation. Therefore, abnormal activation of protein kinases may cause negative effects on normal cell function which may induce tumorigenesis [16]. According to the related theoretical knowledge, protein kinases would be ideal targets for treating hematological malignancies.

### Tyrosine kinase inhibitors (TKIs) in combination with CAR-T cell therapy

T cell receptors (TCR) trigger the activation of T cells. The proximal signaling pathway of TCR is essential in the process of T cell activation. Lymphocyte-specific protein tyrosine kinase (Lck) and proto-oncogene protein tyrosine kinase (Fyn) are members of the Src family kinase, involved in the earliest steps of TCR activation, and Lck deficiency can prevent the conduction of proximal TCR signaling and block T cell development and activation [17]. Therefore, most researchers believe that Lck is more critical in the signaling pathway of TCR than Fyn [18]. Dasatinib, a second-generation BCR-ABL inhibitor, was approved by the FDA in 2006 for the treatment of chronic myelocytic leukemia (CML) [19]. It can inhibit cancer cell proliferation and induce its apoptosis [20, 21]. It can also block the adenosine triphosphate (ATP) binding site of Lck, thereby exerting a powerful ability to inhibit Lck activity. Schade et al. [18] have demonstrated that dasatinib has apparent effects on inhibiting the signal transduction and early activation of T cells in vivo or in vitro and plays a vital part in the subsequent production of chemokines and pro-inflammatory cytokines. Mestermann et al. [22] and Weber et al. [23] demonstrated that dasatinib induces a function-off state in CD4+ and CD8+ CAR-T cells. This state can appear immediately after the application of dasatinib. And it can persist for a few days without influencing the viability of T cells. The function of the CAR-T cells was completely reversible after the removal of the drug. This drug is most likely appropriate for clinically available CAR-T cell pharmacology on/off, allowing doctors to control CAR-T cell function in real time. It was found that dasatinib rapidly governed the CRS of the mice that had received CD19 CAR-T cell therapy, increasing the 48-h survival rate after CRS from 25 to 70%. However, researchers from Zhejiang University recently found that, on the one hand, dasatinib can reduce the differentiation and depletion of CAR-T cells through pharmacological inhibition of T cell activation signals, thereby enhancing their therapeutic effects and persistence in vivo. On the other hand, dasatinib can effectively block or reverse intense activation-induced CAR-T cell differentiation and depletion induced by the stimulation of CD3/CD28 beads or the exposure of antigens, which provides the possibility of clinical application of this drug combined with CAR-T cell therapy [24•]. Recently, Weber et al. [25••] also proposed that treating CAR-T cells with dasatinib attenuates deleterious CAR signaling, which could temporarily block CAR signaling to reverse dysfunction by inducing epigenetic reprogramming in exhausted CAR-T cells. In summary, TKI inhibitors in combination with CAR-T cell therapy seem to be safe and could increase the efficacy and safety of CAR-T cells. Future research directions may be devoted to determining the relationship between dasatinib and epigenetic modification molecules, as well as CAR-T cell activation, differentiation, and exhaustion. We have reasons to believe that dasatinib in combination with CAR-T cell therapy will bring new hope to patients with hematological malignancies. Meanwhile, other second-generation TKIs such as bosutinib and nilotinib, as well as the third-generation TKI ponatinib, may also have the potential to enhance the efficacy of CAR-T cells. Some clinical research about these TKIs applying to patients with Ph+ ALL is undergoing [26]. Based on the promising results of CAR-T cell therapy combined with dasatinib, we anticipate that these TKIs will have significant impact on patients suffering from hematological malignancies, particularly Ph+ ALL.

### Bruton’s tyrosine kinase (BTK) inhibitors in combination with CAR-T cell therapy

A non-receptor kinase called BTK plays a central role in the signaling of an oncogene, making outstanding contributions to cell proliferation and survival in various B cell malignancies [27]. BTK is not only involved in the signaling pathway of the B cell receptor (BCR) but also participates in other signaling pathways of B cells, including chemokine receptors [28], Toll-like receptors (TLRs) [29], and the Fc receptor signaling pathways [30]. There are three kinds of BTK inhibitors on the market, named ibrutinib, acalabrutinib, and zanubrutinib. Studies have shown that CAR-T cells are poorly amplified in vitro from T cells isolated from CLL patients. But when T cells were acquired during ibrutinib treatment, CAR-T had a significantly better expansion [31, 32]. Fan et al. [33] reported that adding ibrutinib during the generation of CD19 CAR-T cells did improve CD19 CAR-T cell production and also enriched less-differentiated naïve-like T cells with low expression of LAG-3, PD-1, and TIM3. Obviously, according to the findings of Fan et al. [33], this novel combination is an extremely promising strategy, but there was no relevant data in vivo to confirm these conclusions in this article. And then, Ruella et al. [34] introduced that ibrutinib improved the response to the treatment of CD19 CAR-T in mantle cell lymphoma (MCL) and enhanced the xenograft MCL mice lifespan compared with CAR-T cell therapy alone. In the same year, the team found that ibrutinib did not impair proliferative capacity, cytotoxicity, or the ability to recognize the tumor of CAR-T cells. Furthermore, ibrutinib could significantly reduce the release of several inflammatory cytokines in NSG mice models, including IL-2, IL-6, IFN-γ, TNF-α, and GM-CSF, as well as the expression of PD-1, LAG-3, TIM-3, and CTLA-4 in CD19 CAR-T cells [35]. Barfi et al.’s findings also suggest that T cell-dependent anti-tumor immune responses can be enhanced by ibrutinib [36, 37]. Moreover, the conclusions regarding T cell cytokine secretion like IFN-γ, IL-2, and TNF-α are similar to those of Qin J.S et al. [38]. Ruella et al. [35] then demonstrated that when MCL cells interacted with different concentrations of ibrutinib in vitro, the level of MIP-1a, MIP-1b, TNF-α, and other tumor proteins was reduced. And this drug indeed did not affect T cell proliferation. Therefore, CAR-T cell therapy combined with ibrutinib could reduce the risk of CRS without affecting the efficacy of CAR-T cell therapy, whether in vitro or in vivo. According to these findings, this team conducted a clinical trial (NCT02640209). Finally, 20 CLL patients were enrolled, and the 48-month overall and progression-free survival rates of 19 out of 20 patients appear to be improved [39]. In addition, a retrospective single-center phase I study of 19 patients with CLL showed that patients treated with ibrutinib and CAR-T cell therapy had lower severity of CRS and lower serum levels of CRS-related cytokines [40•]. Currently, a non-randomized prospective study evaluating ibrutinib combined with CD19 CAR-T cell therapy is underway (NCT03331198). Liu M et al. [41] demonstrated that ibrutinib could weaken the PD-1 expression of CD19 CAR-T both in vitro and in vivo. And then 6 of 7 B-NHL patients achieved CR (ChiCTR-ONN-16009862) after receiving ibrutinib combined with the second-time CD19 CAR-T cell therapy [42]. Qin, J.S et al. [38] suggested that ibrutinib or acalabrutinib intrinsically improved the potential of proliferation or the capacity of survival of CD19 CAR-T cells in CD19+ tumor cells and reduced the cytokine secretion; meanwhile, according to the tumor cell lines used in their experiment which are resistant to the growth inhibition mediated by BTK inhibitors, they thought that pharmacological function of CAR-T cells could be influenced by the efficacy of tumor cell biology mediated by BTK inhibitors. However, more relevant research is needed to verify this conclusion. Meanwhile, adverse effects including bruising/bleeding, cardiovascular damage, skin rash, and diarrhea associated with BTK inhibitors draw our attention [43]. There are some ongoing trials (NCT04234061, NCT04484012) evaluating whether this strategy is safe or efficient. In summary, BTK inhibitors are promising drugs that increase the success rate of CAR-T cell therapy. However, the dose and timing of the BTK inhibitors’ application need further validation.

## Some inhibitors targeting the crucial signaling pathway

When CAR-T cells are infused into the human body, they may cause different degrees of adverse reactions through different signaling pathways in the processes of proliferation, differentiation, and targeted tumor killing. These pathways usually include PI3K-AKT-mTOR and JAK-STAT. Targeting key mediators of signaling pathways and applying drugs to inhibit these pathways may enhance the success rate of CAR-T cell therapy.

### JAK inhibitors in combination with CAR-T cell therapy

JAK-STAT signaling pathway participates in intracellular signal transduction pathways of hematopoietic and immune cells. It can transduce the extracellular signal transmitted by various lymphocytes, growth factors, and cytokines, thus playing a core role in normal hematopoiesis [44–46]. Multiple cytokines and growth factors can activate the JAK family to different degrees, which is critical to the expansion and differentiation of myeloid cells and lymphocytes [47•]. Genes encoding JAK protein kinases, particularly JAK2, are regularly mutated in myeloproliferative neoplasms, which leads to abnormal activation of the JAK/STAT signaling pathway. And this signaling pathway is correlated to the development and survival of cancer cells as well as the progress of chronic inflammation [48]. Ruxolitinib and fedratinib are the two currently FDA-approved JAK2 inhibitors with potent anti-inflammatory and immunosuppressive effects [47•, 49–51]. Kenderian et al. [52] demonstrated that in a mouse xenograft model, ruxolitinib could prevent the progress of severe CRS without compromising CAR-T cells’ anti-tumor effect. Ruxolitinib has been proven to have a good effect in treating severe CRS and excellent tolerability in patients [53]. A patient with Ph+ ALL developed steroid-refractory CRS after receiving sequential CD22/CD19 CAR-T cell infusions. After taking ruxolitinib as adjuvant therapy, the patient’s symptoms improved rapidly. They achieved minimal residual disease-negative CR, which was associated with a reduction in circulating pro-inflammatory marker levels, suggesting that CAR-T cells’ anti-leukemia effects were not affected by this intervention [54]. In another study, fourteen children with r/r B-ALL received infusions with CD19 or CD22 CAR-T cells. And four patients experienced severe (grade ≥3) CRS. They found that serum cytokine levels were significantly decreased by the ruxolitinib intervention, and they all achieved CR 30 days after the infusion. Treatment based on ruxolitinib in two patients with T-ALL also resolved grade 3 CRS induced by CD7 CAR-T cell therapy [55]. Not only could ruxolitinib reduce the levels of cytokines released by other cells in the immune system, it also maintained a certain degree of cytokines that CAR-T cells released. Although CAR-T cells’ proliferation was significantly inhibited, their therapeutic effect was not affected after the withdrawal of ruxolitinib at appropriate doses [56]. What’s more, a selective JAK1 inhibitor called itacitinib was developed to treat graft-versus-host disease. This drug could address CRS with a low risk of immunosuppression and without inhibiting the anti-tumor killing ability or expansion of CAR-T cells in vitro and in mouse lymphoma models. Overall, these results show that itacitinib can be used against CAR-T cell-induced CRS [57]. A phase II clinical trial evaluating itacitinib for the prevention of CRS is currently underway (NCT04071366). However, few studies were focused on the combined application of other kinds of JAK inhibitors and CAR-T cell therapy. According to the above research, JAK inhibitors may not impair the anti-tumor effect of CAR-T, but the potential inhibitory effect of ruxolitinib on the proliferation and maintenance of CAR-T remains a major issue that we need to pay attention to. In addition, hemorrhage and thrombocytopenia are the major common side effects related to ruxolitinib. Though Pan, Jing, et al. found that ruxolitinib was relatively safe during CD19 CAR-T therapy [53], we need more evidence to verify this conclusion. Meanwhile, according to the significant effect of this pathway on the adverse effects of CAR-T cell therapy, this combination regimen, such as ruxolitinib combined with CAR-T cells, is one of the directions for further research to mitigate adverse reactions.

### PI3K inhibitors in combination with CAR-T cell therapy

PI3Kδ inhibitors are one of the most widely studied targeted drugs for treating patients with lymphoma. They cast an essential part in inhibiting tumor progression and reshaping the TME. They have different influences on regulatory T cells (Tregs), myeloid-derived suppressor cells (MDSCs), tumor-associated macrophages (TAMs), etc. [58–61]. Idelalisib, duvelisib, and copanlisib are three kinds of FDA-approved drugs [62–64], of which idelalisib is the first PI3Kδ inhibitor to be approved to treat r/r CLL. Linda et al. [65] found that idelalisib could not only block the signal transduction pathway involved in PI3K but also regulate the differentiation and function of T cells by inhibiting PI3K in vitro and in mouse experiments. Christopher et al. [66] demonstrated that idelalisib increased the quantity and function of T cells and contributed to improving T cells’ qualities during in vitro amplification. These are consistent with the findings of Chellappa and Hanna et al. [60, 67]. Considering the connection of CARs and T cell antigen receptors (TCRs) with the PI3K pathway as well as the differentiation and metabolism of T cells [61], it is feasible to utilize PI3K inhibitors as a means of facilitating the process of producing CAR-T cells. When Sophia et al. added idelalisib to CD19 CAR-T cells in vitro, they found that idelalisib did increase the transduction efficiency of CD19 CAR-T cells by inhibiting PI3Kδ. Meanwhile, it also induced the enrichment of naïve-like T cells with less differentiation and increased the proportion of the lymph node homing marker CD62L, and decreased the expression of PD-1 and TIM-3, thus optimizing the proportion of CAR-T cells with this phenotype. Idelalisib resulted in a CD4:CD8 ratio in chronic lymphocytic leukemia (CLL) patient-derived CAR-T cells that was closer to the ratio in healthy donors [68]. In addition, a study at the 2021 ASH meeting mentioned that during the culture of T cells in vitro, the use of duvelisib, dual inhibition of PI3Kδ/γ, would give priority to the amplification of CD8+ T cells, involving stem cell-like memory T cells and central memory T cells, thereby enhancing CD19 CAR-T cells persistence and cytotoxicity. The expression and epigenetic reprogramming of T cell mitochondrial fusion proteins mitofusins1 and 2 (MFN1/2) were promoted by duvelisib [69]. At the same time, it was found that adding duvelisib to the culture of CAR-T cells could decrease the expression of PD-1, LAG-3, and TIM-3 in CD4+ and CD8+ subsets, increasing the survival rate of mice by enhancing CAR-T cells’ expansion and their anti-CLL efficacy [70]. Funk et al. reconfirmed that when CAR-T cells were exposed to duvelisib, the quantities of memory CD8+ T cells with stem cell properties in CAR-T products were increased, giving CAR-T cells an epigenetic pathway in vivo with greater amplification and anti-tumor activity [71••]. Now, research on CAR-T cell therapy with PI3K inhibitors to treat hematological malignancies is limited. More relevant data from pre-clinical and clinical trials are required to confirm the influences on CAR-T cell therapy further.

### mTOR inhibitors in combination with CAR-T cell therapy

By analyzing 52 FDA-approved kinase inhibitors, researchers discovered that mTOR inhibitors could influence the proliferation of T cells and relevant cytokine secretion after the activation of TCR [72]**.** In addition, the PI3K/Akt/mTOR signaling plays a vital part in the cell cycle. Researchers have found that CAR-T cells’ anti-tumor activity will increase due to the inhibition of Akt signaling during the preparation process of CAR-T cells, suggesting that the inhibition of this signaling may be another therapeutic target to enhance the anti-tumor activity of CAR-T cell therapy [73]. Meanwhile, sirolimus, temsirolimus, and everolimus are mTOR inhibitors that are FDA-approved. A recent study showed that they could impair cytokine secretion associated with CD19-TCB without reducing its anti-tumor efficacy at the proper doses, whether in vivo or in lymphoma huNSG mice. Based on these data, they thought that mTOR inhibitors might be a better option to prevent the development of CRS without interfering with T cell killing cells compared with JAK, Src inhibitors, and dexamethasone [72]. In fact, mTOR inhibitors are widely applied in different solid cancers due to their anti-tumor activity, combined with a TCB that targets solid tumors, which seems to be a way to prevent the occurrence of CRS while retaining its efficacy [74, 75]. However, the signaling of mTOR is often dysregulated in different cancers, such as breast, prostate, lung, liver, and renal cell carcinomas. Studies showed that the upregulation of mTOR signaling may promote growth factor receptor signaling, angiogenesis, glycolytic activity, lipid metabolism, cancer cell migration, and the suppression of autophagy, leading to tumor growth and progression [76, 77]. Esfahani et al. [78] found that sirolimus could change the immune landscape and promote patients with renal transplant tolerance while maintaining anti-tumor activity mediated by pembrolizumab. What’s more, rapamycin (Rapa) has direct anti-tumor activity, but at the same time it can inhibit effector T cells, so it may also have an inhibitory effect on CAR-T cells. Based on this, Huye et al. [79] developed rapamycin-resistant CD19 CAR-T cells and found that the anti-tumor activity was increased in Burkitt’s lymphoma and ALL cell lines. All in all, these results support the idea that targeting the mTOR pathway is a new way to reduce the occurrence of CRS associated with immunotherapies and promote the development of CAR-T cell therapy.

## Apoptosis regulators

Apoptosis has been a hot topic in recent years, which is of great significance in the occurrence and development of many diseases. Tumors usually upregulate anti-apoptotic proteins or silence pro-apoptotic proteins resulting in an imbalance of apoptosis [80, 81]. CAR-T cells kill tumor cells via apoptosis induction. The application of apoptosis inhibitors to re-sensitize tumor cells to apoptosis before CAR-T cell therapy is an attractive strategy.

### Bcl-2 inhibitors in combination with CAR-T cell therapy

B cell lymphoma-2 (Bcl-2) is a proto-oncogene located on human chromosome 18q21. In multivariate analysis, Bcl-2 protein level was the most significant predictor of patient overall survival. The reduced apoptotic potential and enhanced accumulation of leukemia cells were related to the level of its expression [82]. Therefore, inhibiting the overexpression of the Bcl-2 family members plays an essential role in the development of tumors and is also a strategy of combination therapy. Studies in vitro showed that when tumor cells were pre-sensitized by a Bcl-2 inhibitor called venetoclax, CD19 CAR-T cells’ killing efficiency was significantly increased. And the early proliferation, persistence, resistance to immune escape, and anti-tumor efficacy of CD19 CAR-T cells were enhanced in this way [83]. Recently, a team exploited a unique CAR construct by integrating Bcl-2 into CAR-T cells and found that this strategy could enhance CAR-T cells’ proliferation, thereby enhancing the anti-tumor activity in xenografted lymphoma mice and prolonging their lifespan. This finding provides new ideas for optimizing CAR-T cell therapy in anti-lymphoma strategies [84]. Navitoclax, a novel Bcl-2 inhibitor, exhibits cytotoxic activity in myeloproliferative neoplasm (MPN)-derived cell lines and in vitro specimens. Apoptosis of tumor cells was significantly increased when combined with both CAR-T cells and navitoclax, or with navitoclax as a pre-sensitizer [85]. Researchers should conduct more studies to explore the molecular mechanisms associated with the role of navitoclax in hematologic tumors (except ALL) [86].

## Epigenetic modulators

Epigenetics is a discipline that mainly focuses on the study of gene transcription and altered translation activity mediated by DNA methylation, histone modifications, chromosome remodeling, RNA, and RNA modifications [87]. In recent years, researchers have discovered that mechanisms related to epigenetic modifications of the genome (e.g., DNA methylation and histone modifications) may lead to impaired signaling in normal hematopoietic pathways. Therefore, epigenetic modifications are considered important targets for the therapy of leukemia and other hematological malignancies [88–91]. Epigenetic drugs typically act on enzymes essential for epigenetic modifications, with the main strategies being the inhibition of DNA methyltransferases and histone deacetylases (HDACs). Below, we will discuss the most widely used HDAC inhibitors and DNA demethylation drugs in clinical practice at present.

### HDAC inhibitors in combination with CAR-T cell therapy

Histone acetyltransferases (HATs) and HDACs regulate histone acetylation, thereby regulating gene expression. The imbalance of histone acetylation causes the aberrant expression of genes, which also activates oncogenes, inactivates tumor suppressors, inhibits the programmed death of cells, mediates the dysregulation of immunity, and ultimately arouses the progression of tumors [92]. Several studies have shown that HDAC inhibitors (HDACi) can improve the expression levels of mRNA and protein in CD20 in Burkitt lymphoma, thereby increasing the expression of CD20 in cancer cells [93–95]. A report further revealed that HDACi upregulated the expression of CD20 in cancer cells by increasing the acetylation level of H3K9 in the CD20 promoter region. At the same time, the team discovered that after pretreatment with HDACi for 48 h, CD20 CAR-T cells secreted more IFN-γ and TNF-α and that HDACi could enhance the cytotoxic activity of CD20 CAR-T cells against Burkitt lymphoma cells in vitro and in vivo [96]. However, this team did not testify whether the secretion of IFN-γ and TNF-α influenced the development of CRS. Some papers have described that CAR-NK cell function could be improved by HDACi [97, 98]. Romidepsin is an active HDACi that could not only enhance NKG2D ligand expression in cancer cells but also activate NKG2D expression in NK cells, so when Burkitt lymphoma cells were treated with romidepsin in advance, they would be easier to kill by CD20 CAR-NK cells. In humanized Raji xenografted NSG mice, the combination of the two achieved a better therapeutic effect than monotherapy [98]. A recent study reconfirmed that this drug increased NK cells’ expansion and improved CD20 CAR-NK activity no matter in vivo or in vitro, providing an experimental basis for the combination of romidepsin and CD20 CAR-NK to treat CD20+ Burkitt lymphoma [97]. Torre et al. [99] found that when human non-Hodgkin’s lymphoma cell lines responded poorly to CD19 CAR-T cell therapy gradually, HDACi (vorinostat, also known as SAHA or panobinostat) reversed the resistance to CD19 CAR-T cell therapy. But we have not known the specific molecular mechanism until now. In addition, some studies have shown that patients with B-ALL who have failed after CD19 CAR-T therapy can receive CD22 CAR-T cell therapy, which displays high efficacy in disease remission. However, the low density of target antigens is still a barrier to CAR-T cell therapy among many limitations [100, 101]. Chidamide is a novel oral selective HDACi initially developed in China. Tumor killing mediated by NK cells and antigen-specific cytotoxic T cells can be induced and enhanced by chidamide. A recent study showed that chidamide upregulated the density of CD22 in B cell tumor lines and primary cells, thereby strengthening the curative effect of CD22 CAR-T cell therapy [102]. Based on these studies, we are looking forward to the promising results that HDACi combined with CAR-T cell therapy provides in AML because of the antigen density improvement.

### Demethylating drugs in combination with CAR-T cell therapy

DNA methylation has been shown to promote T cell depletion, and aberrant DNA methylation plays a pivotal role in tumor development and progression [103, 104]. Azacitidine (AZA) and decitabine (DAC), two FDA-approved DNA demethylating drugs, have been shown to invert the DNA methylation programs correlated with exhaustion, causing tumor cell reprogramming and improving T cell responses to tumors [103]. However, these drugs may lead to the occurrence of pancytopenia [105], which may increase the risk of infection in patients after CAR-T cells’ infusion. The number of CFU-GM colonies in healthy donor BMMC did not decrease further after being sub-treated with AZA and CD123 CAR-T, suggesting that the combination of the two is unlikely to cause severe hematopoietic insufficiency [106••]. In addition, pretreatment of AML cells with AZA before the application of CD123 CAR-T cells promoted the upregulation of tumor cell target antigen expression, meanwhile enhancing the anti-tumor efficacy of CD123 CAR-T cells, and prolonging the survival time of AML mice models. One team constructing CD70 CAR-T for AML found that when AML exposure to AZA at physiologically dose-dependent concentrations increased the expression of CD70 antigen in tumor cells and enhanced the efficacy of CD70 CAR-T, then they demonstrated this in mice models [107]. Zebley et al. [108] analyzed serial clinical samples from patients with ALL and revealed that CD8+ CD19 CAR-T cells underwent DNA methylation reprogramming after infusion, leading to depletion of cell differentiation. Wang, Y et al. [109••] pre-treated CD19 CAR-T cells with low-dose DAC (10nM) and found that DAC had a demethylating effect on CAR-T cells, reduced CAR-T cell depletion, and enhanced CAR-T proliferation and anti-tumor function in vitro and in mouse models. This is consistent with the results of Li et al. [110]. After treatment with DAC, they found that lymphoma cells were more susceptible to being killed by CD19 CAR-T cells because of the increased expression of surface antigen density, and two patients with r/r lymphoma treated with DAC and CAR-T cells both achieved CR. It shows that this combination is feasible. You, L et al. [111] found that pretreatment of CD123 CAR-T cells with ultra-low-dose DAC (0.1–1μM) increased CD123 CAR-T cells activation and the anti-leukemic effect increasing in vivo. In addition, Qu et al. [112] found that six patients achieved molecular CR with DAC as maintenance therapy after CAR-T therapy, indicating that the application of DAC may improve the prognosis of r/r AL patients with TP53 alterations after receiving CAR-T therapy. However, due to the small sample of cases, multiple variables, and lack of homogeneity between groups, the results are yet to be validated again in the future. Overall, the selections of CAR-T targets, the choices of demethylating drugs, and the mode and timing of administration still need to be deliberated regarding the combination of demethylating drugs with CAR-T. Based on the current pre-clinical reports, it is reasonable to expect that the combination of these two drugs will bring benefits to patients with hematological malignancies.

## Immunomodulatory agents in combination with CAR-T cell therapy

Immunomodulatory drugs (IMiDs) such as thalidomide, and its derivatives lenalidomide and pomalidomide, have been widely used in cancer and autoimmune diseases [113]. Because these drugs can directly impair myeloma cells’ growth and facilitate the anti-tumor immune responses. At present, they are mainly used in multiple myeloma (MM). Moreover, IMiDs can affect the proliferation, differentiation, and function of T cells [114–117]. So the combination of CAR-T therapy with these drugs may improve the prognosis of patients with multiple myeloma (MM). Works et al. [118] found that when lenalidomide was combined with BCMA CAR-T, lenalidomide could increase cytokine secretion (IL-2, IFN-γ, and TNF-α) as well as the cytolytic activity of BCMA CAR-T therapy in a concentration-dependent manner. The count of CAR-T cells in mice’s peripheral blood was improved in the presence of lenalidomide, and the survival of mice has also been enhanced. A 51-year-old man with MM received lenalidomide the day before BCMA CAR-T therapy and achieved very good PR lasting more than 8 months. This case demonstrated that this combination therapy is feasible and effective [119]. In addition, because CS1 is highly selectively expressed in MM cells and rarely expressed in other kinds of cells, CS1 CAR-T cells were prepared based on treatment targeting MM [120, 121]. The addition of lenalidomide to CS1 CAR-T cells in vitro and in vivo experiments was found to improve the formation of immune synapses between CS1 CAR-T cells and tumor cells, improve the function and persistence of CAR-T, and increase its anti-tumor activity [121]. However, this strategy needs to be supported by relevant clinical data. Currently, there are four ongoing clinical trials of CS1 CAR-T for MM (NCT03710421, NCT04541368, NCT03778346, and NCT04662099).

## Cytokines in combination with CAR-T cell therapy

Cytokines are molecular messengers that enhance the function of the immune system and enable cells to transmit information to each other. Different kinds of cytokines play various roles in enhancing anti-tumor efficiency through the immune system [122]. However, the toxicity and possibility of promoting the development of tumors associated with cytokines are issues that should be carefully considered in the specific application. Taking advantage of the favorable nature of cytokines and combining them with immunotherapy might be a promising strategy. It is well known that during the preparation or design of CAR-T cells, the addition of cytokines such as IL-2, IL-4, IL-7, IL-15, and IL-21 can promote the growth of CAR-T cells [123–126]. These cytokines can promote T cell growth, survival, and expansion while providing resistance to immune suppression. So far, IL-2, IL-7 [127], IL-9 [128], IL-12 [129], IL-13 [130], IL-15 [131], IL-18 [132], IL-21 [133], IL-23 [134], IL-33 [135], and other cytokines have been used in pre-clinical studies in conjugations with CAR-T cells, while some cytokines, such as IL-1 [136, 137], IL-6 [138, 139], IL-10 [140], TNF-α [136, 141], IFN-γ [138], and GM-CSF [142], have some negative effects on CAR-T cell therapy participating in the development of CRS during CAR-T cell therapy. Due to further research on the mechanisms of CRS and ICANS, as well as the continuous exploitation and validation of related cytokine inhibitors, concrete results have been achieved in the treatment of adverse reactions during CAR-T cell therapy. We hope that we will control negative responses in time to improve the success rate of CAR-T cell therapy as possible as we can.

## Some other kinds of small-molecule inhibitors in combination with CAR-T cell therapy

In addition to the drugs mentioned above, cyclooxygenase-2 inhibitors, γ-secretase inhibitors (GSIs), proteasome inhibitors, selective nuclear export inhibitors, Akt inhibitors, etc. have also been studied in combination with CAR-T cell therapy by researchers. Cyclooxygenase-2 (COX-2), an enzyme induced by inflammatory and mitotic stimuli, enhances prostaglandin synthesis in inflammatory and tumor tissues [143]. Due to the anti-inflammatory effects of COX inhibitors, they have been recommended to treat tumors to inhibit the levels of inflammatory factors in TME, which can promote the expansion, survival, and migration of tumor cells [144, 145]. Previous studies have shown that COX inhibitors inhibited normal T cells [146]. Therefore, recently, a team investigated the effects when CD19 CAR-T cells were combined with various concentrations of COX inhibitors (celecoxib and aspirin) would happen. They found that NSAIDs inhibited the expression of PD-1 and TIM-3, induced apoptosis in CD19 CAR-T cells, and influenced CAR-T cells’ proliferation. Thus, just like every coin has two sides, the application of COX inhibitors shows some specific anti-tumor effects, but it impairs CD19 CAR-T cells’ quantity and quality [147], so these kinds of drugs should be used cautiously [148]. Pont et al. [149] found that when MM cell lines were co-cultured with gamma-secretase inhibitors (GSIs), the expression of BCMA on the cell surface increased three- to fivefold. The team has initiated a phase I clinical trial combining BCMA CAR-T cell therapy with GSIs (NCT03502577). Notably, when BCMA expression levels on target cells were low, applying GSIs would enhance their density, but not when BCMA expression levels were already high. Another study also supported the view of Pont et al. [150]. Based on these results, patients receiving BCMA CAR-T cell therapy and GSIs together will have a better prognosis, and to some extent, this strategy can prevent the relapse of MM due to low antigen-expressing cells. Bortezomib, the first proteasome inhibitor, was approved for treating MM. Ixazomib and carfilzomib are the new generations of proteasome inhibitors. The safety and efficacy of bortezomib combined with chemotherapy in r/r ALL have been demonstrated in the pediatric population, but the role of proteasome inhibitors playing in adult r/r ALL needs more related studies to verify [151]. At present, there are few strategies for novel proteasome inhibitors and their combination with CAR-T cell therapy applying in r/r ALL, so it is necessary for further research and exploration. Exportin 1 (XPO1), a nucleocytoplasmic shuttle protein, contributes to exporting proteins from the nucleus to the cytoplasm [152]. Selinexor and eltanexor, the selective inhibitors of nuclear export (SINEs) of XPO1, induce apoptosis in tumor cells by promoting the accumulation of tumor suppressor proteins in the nucleus, which has achieved success in treating hematological malignancies currently [153–155]. When these two SINEs were combined with CAR-T cell therapy simultaneously, they were toxic to CAR-T cells. They hampered CAR-T cells’ functions by affecting the capacity of cytokines released and their cytotoxicity. But when tumor cells were pre-treated with eltanexor, the expressions of PD-1, TIM-3, and LAG-3 of CAR-T cells were decreased, and the cytotoxicity improved. Thus, scholars expect that the sequential use of SINE and CAR-T cell therapy can promote the anti-tumor ability of the latter [156].

## Discussion

**Fig. 3:**
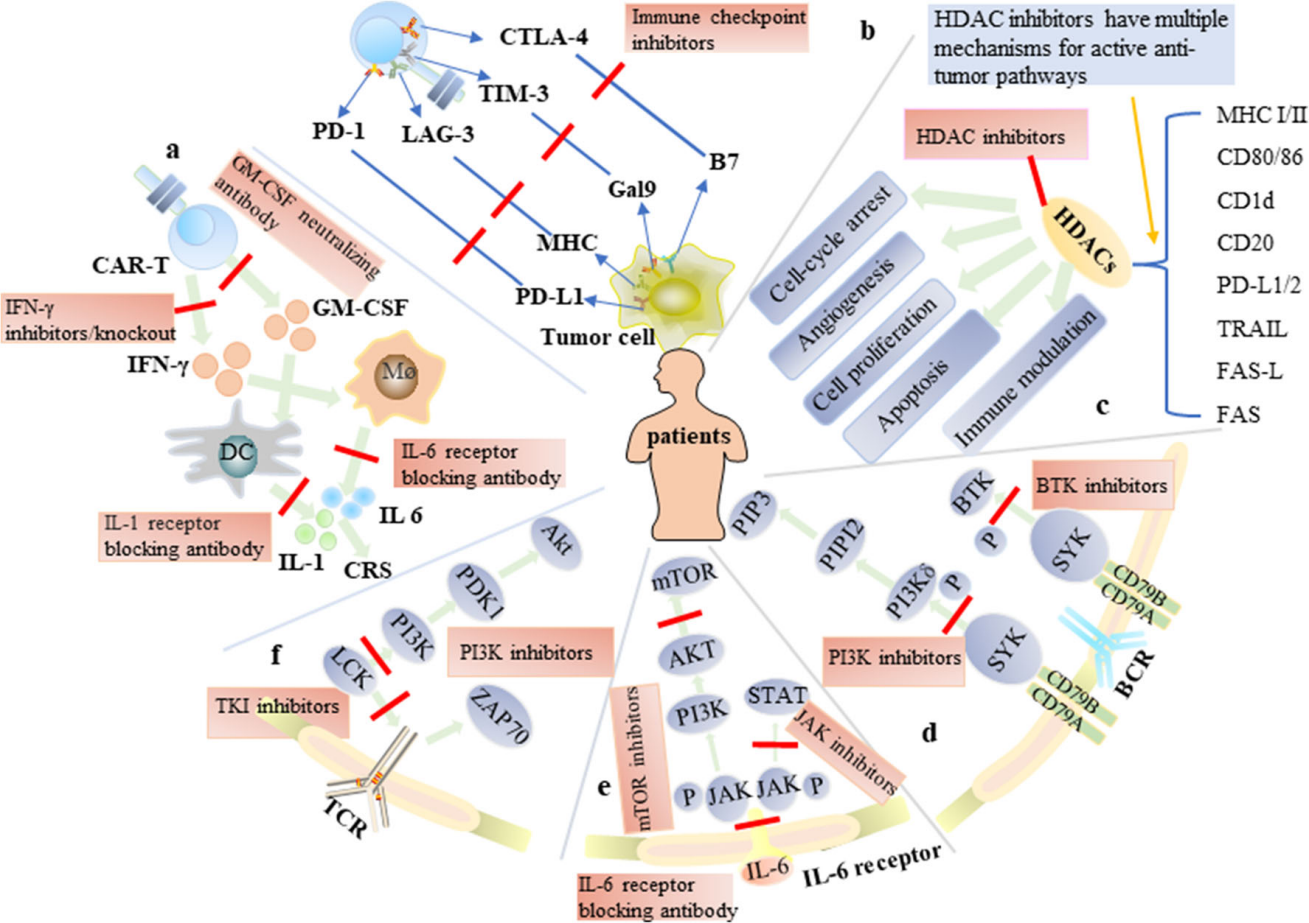
Mechanisms of action of small-molecule inhibitors. (**a**) Current acknowledgment and intervention of CRS induced by CAR-T cells. (**b**) Inhibitory TCRs and their ligand, including LAG3-MHC, CTLA4-B7, PD1-PDL1, and TIM3-Gal9. CTLA 4, cytotoxic T-lymphocyte-associated protein 4; MHC, major histocompatibility complex; LAG3, lymphocyte-activation gene 3; PD-1, programmed cell death protein 1; PD-L1, programmed death-ligand 1; TIM-3, T cell immunoglobulin, and mucin domain-containing protein 3; TCR, T cell receptor; Gal9, galectin-9. (**c**) The main mechanism of action of HDAC and HDACis in B cell lymphoma. (**d**) BCR signaling and relevant inhibitors. (**e**) IL-6/JAK/STAT signaling and relevant inhibitors. (**f**) TCR signaling and relevant inhibitors. (a short red line indicates inhibition or blocking.)

Asides from continuous modification of CAR-T cells, more and more research results show that small-molecule compounds make significant contributions to the collaborative treatment of hematological malignancies with CAR-T cell therapy, demonstrating that these combined strategies could overcome many limitations in the current CAR-T cell therapy (Fig. 3). TKIs, for instance, have been shown to act as inhibitors in the signaling pathways of TCR and the cytotoxicity of CAR-T cells. Dasatinib, in particular, has the potential to control the functions of CAR-T cells like a switch, improving their safety, persistence, and efficacy. TKIs, BTK inhibitors, cytokine receptor antagonists, JAK inhibitors, mTOR inhibitors, etc. can decrease the probability of CRS occurrence during CAR-T cell therapy, prevent adverse reactions, and improve the tolerance rates of patients. HDACis, γ-secretase inhibitors, and demethylating agents can upregulate the density of tumor antigens on target cells’ surfaces, and reduce tumor immune escape and disease recurrence, consequently increasing CAR-T cell therapy’s success rates. In pre-clinical studies, when tumor cells were pre-treated with Bcl-2 inhibitors, these cells were more easily killed by CAR-T cells, but studies associated with this result are limited. By the way, the transduction efficiency of CAR-T cells was increased by PI3K inhibitors, which were applied in advance by researchers, but similar to the cases of Bcl-2 inhibitors, there are fewer related studies, so hard pieces of evidence are lacking. There is a specific bidirectional effect for COX-2 inhibitors and SINE in treating tumors with CAR-T cell therapy, which requires further research to achieve a balance. Moreover, there are also studies about immunomodulatory drugs, proteasome inhibitors, etc., combined with CAR-T cell therapy, to some extent they could increase the anti-tumor activity of CAR-T cells (Table 1).

**Table 1:** Relevant trials about small-molecule compounds combined with CAR-T cell therapy for treating hematological malignancies

**Combination drugs**	**Drugs name**	**Types of CAR-T**	**Diseases**	**Results**
**TKIs**	Dasatinib [22]	Anti-CD19 CAR-T	Burkitt’s lymphoma (pre-clinical trial)	Cytolytic activity and cytokine production were halted. The proliferation of CAR-T was promoted.
	Dasatinib [23]	Anti-CD19 CAR-T	B-ALL (pre-clinical trial)	Reversibly suppressed the cytotoxicity, cytokine secretion, and proliferation of CAR-T.
	Dasatinib [24•]	Anti-CD19 CAR-T	B-ALL (pre-clinical trial)	Reduced CAR-T differentiation and exhaustion, enhanced their therapeutic efficacy and persistence.
	Dasatinib [25••]	Anti-CD19 CAR-T	B-ALL (pre-clinical trial)	Reversed dysfunction and induced epigenetic reprogramming in exhausted CAR-T.
**BTK inhibitors**	Ibrutinib [31]	Anti-CD19 CAR-T	CLL (pre-clinical trial)	Enhanced the generation of CAR-T, and improved their engraftment and therapeutic efficacy.
	Ibrutinib [33]	Anti-CD19 CAR-T	CLL (pre-clinical trial)	Increased CAR-T yields and enriches CAR-T with less-differentiated phenotypes and lower expression of exhaustion markers.
	Ibrutinib [34]	Anti-CD19 CAR-T	MCL (pre-clinical trial)	Enhanced anti-tumor activity.
	Ibrutinib [35]	Anti-CD19 CAR-T	MCL (pre-clinical trial)	Reduced CRS and prolongs survival without impairing CAR-T proliferation.
	Ibrutinib/acalabrutinib [38]	Anti-CD19 CAR-T	CD19^+^ tumor cells (pre-clinical trial)	Improved CAR-T cells’ proliferation or survival and reduced cytokine secretion.
	Ibrutinib [39]	Anti-CD19 CAR-T	CLL (clinical trial: NCT02640209)	Twenty CLL patients received ibrutinib, and then, nineteen received CAR-T, achieving deep remissions.
	Ibrutinib [40•]	Anti-CD19 CAR-T	CLL (clinical trial: NCT01865617)	Nineteen r/r CLL patients were well tolerated, with low CRS severity and high response rates.
	Ibrutinib [41]	Anti-CD19 CAR-T	Lymphoma (pre-clinical trial)	Influenced the TME to play the synergistic effect with CAR-T.
	Ibrutinib [42]	Anti-CD19 AR-T	B-NHL (clinical trial: ChiCTR-ONN-16009862)	Seven B-NHL patients were involved; the CRS grades and hematological toxicity grades were higher but could control. Six achieved CR; one achieved PR.
**JAK inhibitors**	Ruxolitinib [53]	Anti-CD19/CD22 CAR-T	r/r B-ALL (clinical trial: 20191025-PJ-001)	Fourteen children with r/r B-ALL were involved, having a rapid resolution of steroid-refractory and life-threatening CRS during CAR-T therapy.
	Ruxolitinib [54]	Anti-CD19/CD22 CAR-T	r/r B-ALL (clinical trial)	One patient was involved, reaching MRD negative CR, and CRS symptoms improved rapidly.
	Ruxolitinib [55]	Anti-CD7 CAR-T	T-ALL (clinical trial: ChiCTR190002531/ISRCTN19144142)	Two patients were involved. Both achieved CR. Life-threatening CRS was mitigated without impacting CAR-T target-specific cytotoxicity and expansions.
	Ruxolitinib [56]	Anti-CD19 CAR-T	Burkitt’s lymphoma (pre-clinical trial)	Inhibited CAR-T proliferation and reduced cytokine levels without damaging viability.
	Itacitinib [57]	Anti-CD19 CAR-T	B-ALL (pre-clinical trial)	Prevents CAR-T-associated CRS without negatively affecting anti-tumor efficacy.
**PI3K inhibitors**	Idelalisib [68]	Anti-CD19 CAR-T	CLL (pre-clinical trial)	Increased transduction efficiency, and improved the composition of CAR-T without impairing cells’ viability and expansion.
	Duvelisib [69]	Anti-CD19 CAR-T	CLL (pre-clinical trial)	Promoted mitochondrial fusion and epigenetic reprogramming of T cells.
	Duvelisib [70]	Anti-CD19 CAR-T	CLL (pre-clinical trial)	Decreased the expression of immune checkpoint molecules and enhanced proliferation and anti-tumor activity of CAR-T.
	Duvelisib [71••]	Anti-CD19 CAR-T	CLL (pre-clinical trial)	Enriched T-stem cell memory CD8^+^ CAR-T, altered epigenetic pathways, enhanced expansion, and anti-tumor activity.
**Akt inhibitors**	[73]	Anti-CD19 CAR-T	B-ALL (pre-clinical trial)	CAR-T displayed comparatively higher anti-tumor activity.
**mTOR inhibitors**	Rapamycin (Rapa) [79]	Anti-CD19 CAR-T	Burkitt’s lymphoma/B-ALL (pre-clinical trial)	Produced greater anti-tumor activity.
**Bcl-2 inhibitors**	Venetoclax [83]	Anti-CD19 CAR-T	Burkitt’s lymphoma (pre-clinical trial)	Strengthened the anti-tumor activity, and enhanced the persistence of CAR-T*.*
	Navitoclax [85]	Anti-CD19 CAR-T	Lymphoma (pre-clinical trial)	Increased the apoptosis of tumor cells, and improved the efficacy of CAR-T.
**HDAC inhibitors**	Romidepsin [96]	Anti-CD20 CAR-T	Burkitt’s lymphoma/B-ALL (pre-clinical trial)	Enhanced the cytotoxic effect of CAR-T.
	Romidepsin [97]	Anti-CD20 CAR-NK	Burkitt’s lymphoma (pre-clinical trial)	Enhanced the activity of CD20 CAR-NK.
	Panobinostat [99]	Anti-CD19 CAR-T	NHL (pre-clinical trial)	Reversed the resistance of R-NHL to the cytotoxic effects of anti-CD19 CAR CTLs and rhTRAIL.
	Chidamide [102]	Anti-CD22 CAR-T	B-ALL (pre-clinical trial)	Increased the expression of CD22 on the cell surface. Enhanced the efficacy of CD22 CAR-T.
**Demethylating drugs**	Azacitidine [106••]	Anti-CD123 CAR-T	AML (pre-clinical trial)	Increased CD123 expression of the CAR target and enhanced anti-tumor effects.
	Azacitidine [107]	Anti-CD70 CAR-T	AML (pre-clinical trial)	Increased CD70 expression of the CAR target and enhanced CAR-T function.
	Decitabine [109••]	Anti-CD19 CAR-T	Burkitt’s lymphoma/B-ALL (pre-clinical trial)	Enhanced CAR-T cell proliferation, reduced CAR-T cell depletion, and increased anti-tumor function.
	Decitabine [110]	Anti-CD19 CAR-T	B cell malignancies (pre-clinical/clinical trial: NCT02851589)	Increased CD19 antigen expression and did not influence the function of CAR-T cells in vitro, and in vivo both two patients achieved CR.
	Decitabine [111]	Anti-CD123 CAR-T	AML (pre-clinical trial)	Enhanced the function of CD123 CAR-T cells both in vivo and in vitro.
	Decitabine [112]	CAR-T	r/r AL patients with TP53 alterations. (clinical trial: NCT03919240, NCT03614858, NCT03896854)	11 patients were involved (9 B-ALL, 1 AML, and 1 B-lymphoid/myeloid biphenotypic mixed phenotype AL). 6 patients received decitabine and received mCR; this drug might improve the outcome of CAR-T.
**IMiDs**	Lenalidomide [118]	Anti-BCMA CAR-T	MM (pre-clinical trial)	Enhanced the function of CAR-T, increased cytokine secretion, and cytolytic activity.
	Lenalidomide [119]	Anti-BCMA CAR-T	MM (clinical trial: NCT04003168)	1 IgD-λ man involved, achieved very good PR lasting more than 8 months.
	Lenalidomide [121]	Anti-CS1 CAR-T	MM (pre-clinical trial)	Enhanced anti-tumor activity and prolonged CAR-T cells’ persistence.
**COX-2 inhibitors**	Celecoxib [148]	Anti-CD19 CAR-T	Burkitt’s lymphoma (pre-clinical trial)	Hampered the quantity and quality of CD19 CAR-T in a dose-dependent manner.
**γ-Secretase inhibitors**	[149]	Anti-BCMA CAR-T	MM (pre-clinical trial)	Increased BCMA surface expression on myeloma cells. Enhanced CAR-T efficacy.
	[150]	Anti-BCMA CAR-T	MM (pre-clinical trial)	Increased the recognition of cancer cells by CAR-T.
**SINES**	Eltanexor and Selinexor [156]	Anti-CD19 CAR-T	Burkitt’s lymphoma/B-ALL (pre-clinical trial)	Decreased the release of cytokines and enhanced the function of CAR-T.

*TKI*, tyrosine kinase inhibitor; *BTK*, Bruton tyrosine kinase; *ICI*, immune checkpoint inhibitor; *Bcl-2*, B cell lymphoma-2; *IL-6*, interleukin-6; *IL-1*, interleukin-1; *GM-CSF*, granulocyte-macrophage colony-stimulating factor; *COX-2*, cyclooxygenase-2; *HDAC*, histone deacetylase; *SINES*, selective inhibitors of nuclear export; *Akt*, protein kinase B; *mTOR*, mammalian target of rapamycin; *CLL*, chronic lymphocytic leukemia; *ALL*, acute lymphoblastic leukemia; *r/r B-ALL*, refractory and relapsed B cell acute lymphoblastic leukemia; *MCL*, mantle cell lymphoma; *B-NHL*, B cell non-Hodgkin lymphoma; *r/r MM*, refractory/relapsed multiple myeloma; *CRS*, cytokine release syndrome; *DLBCL*, diffuse large B cell lymphoma; *R-NHL*, resistant non-Hodgkin’s lymphoma sublines; *rhTRAIL*, tumor necrosis factor-related apoptosis-inducing ligand; *CR*, complete response; *OR*, objective response; *PR*, partial response; *MRD*, minimal residual disease

## Conclusion

Overall, the efficacy of CAR-T cell therapy can be further improved to a large extent by the combination of small-molecule compounds. This way can also reduce adverse reactions, improve the tolerance of patients, and then increase the success rates of CAR-T cell therapy. These drugs are like boosters in CAR-T cell therapy, just like a role that the icing plays on the cake. Although many small-molecule compounds are mentioned in this article, there is no complete detailed list, and many related clinical trials are underway. By the way, in addition to small-molecule compounds, CAR-T cell therapy combined with monoclonal antibodies (PD-1/PD-L1, obinutuzumab, rituximab, blinatumomab, daratumumab, etc.), and drugs related to cell metabolism (etoposide, cyclophosphamide, etc.) and so on is also worthy of exploring. Especially immune checkpoint inhibitors which belong to monoclonal antibodies can not only overcome the inhibition of TME but also improve CAR-T cells’ proliferation, which may have a synergistic effect with CAR-T cell therapy [157, 158]. Combined immunotherapy for hematological malignancies should be explored further in order to improve the anti-tumor efficacy and reduce side effects when compared to these treatments alone. In addition, there are some other strategies, such as localized radiotherapy and oncolytic viruses. We eagerly await the results of future studies.

## Supplementary Information

ESM 1 (DOC 26 kb)

## Data Availability

Not applicable
