# Supplementary material for: Small-Molecule Compounds Boost CAR-T Cell Therapy in Hematological Malignancies
Source: Curr Treat Options Oncol. 2023 Jan 26;24(3):184–211. doi: 10.1007/s11864-023-01049-4 (PMC9992085; doi:10.1007/s11864-023-01049-4)
Supplement: Supplementary file 1 — (DOC 26 kb) [file 11864_2023_1049_MOESM1_ESM.doc]

All the figures and tables we have included are originals. They don’t need to obtain any permission from other sources in order to use.
